# Performance Evaluation of Sinusoidal Power Supplies for Ozone Generation in Water Purification Applications

**DOI:** 10.3390/s24134063

**Published:** 2024-06-22

**Authors:** Mostafa M. Shibl, Omar Shouman, Ahmed Mahmoud, Ahmed M. Massoud

**Affiliations:** Department of Electrical Engineering, Qatar University, Doha 2713, Qatar; os1902986@qu.edu.qa (O.S.); am1900927@qu.edu.qa (A.M.); ahmed.massoud@qu.edu.qa (A.M.M.)

**Keywords:** ozone generators, sinusoidal power supplies, passive filters, resonance, water purification, ozone chamber

## Abstract

Ozone generation is a water disinfection method, superior to chlorine in terms of fewer byproducts and no residual taste. However, its high production cost limits its widespread adoption. This paper designs an ozone generation sinusoidal power supply for water treatment. Ozone generation requires a high-frequency and high-voltage power supply to produce ozone from oxygen molecules. The study evaluates two power supply topologies, one with a parallel LC filter and the other with an LCL filter, assessing their feasibility, effectiveness, and reliability. Theoretically, the LCL filter achieves higher gains than the parallel LC filter. The larger inductance in the parallel LC filter reduces gain, while the larger inductance in the LCL filter increases gain. Simulation and practical results validate these findings, achieving gains of 40 for the parallel LC filter and 150 for the LCL filter.

## 1. Introduction

According to a report by WaterAid, 771 million people do not have access to clean water. This causes the death of a newborn every minute due to infections from a lack of clean water [1]. Furthermore, more than 2 billion people use contaminated drinking water, which leads to approximately 700,000 yearly deaths related to pathogens in drinking water [2]. In addition, the global demand for freshwater use has increased exponentially since the beginning of the 20th century [3]. Additionally, the lack of clean water causes a lack of hygiene, such that 2.3 billion people lack basic hygiene facilities, including 670 million people who do not have handwashing facilities [4].

Thus, nowadays, many technological advancements are responsible for a more sustainable future, especially after the release of the Sustainable Development Goals (SDGs) in 2015 [5] by the United Nations. A clean water supply contributes to two goals: clean water and sanitation (goal 6) and good health and well-being (goal 3). Moreover, another driving factor for developing water purification (WP) methods is the importance of always having a secure clean water supply, despite external factors such as water intoxication. Thus, the increased importance of having a clean water supply in all countries has increased research efforts in the pursuit of finding WP methods that are simple, effective, and cheap. However, despite efforts to find an effective WP method, advances in clean-water-related services have been slow [5].

Approximately 75% of the Earth’s surface is water. However, only 2% of this water is clean freshwater because most of the Earth’s surface is covered by oceans and seas. Previously, many WP methods have allowed the use of the Earth’s water sources, and these have their benefits and drawbacks. WP methods such as boiling, sedimentation and decantation, and solar disinfection are considered old and ineffective due to the destruction of mineral salts in the water, the inadequate removal of soluble pathogens, and the inefficiency when used for large volumes of water. This has led to chlorination being the most adopted WP method since the beginning of the 20th century because of its inexpensiveness and availability. However, the disadvantages of chlorination and its reported health issues have caused a change in global WP trends and the discovery of new technologies, such as the utilization of ozone for WP.

**Figure 1:**
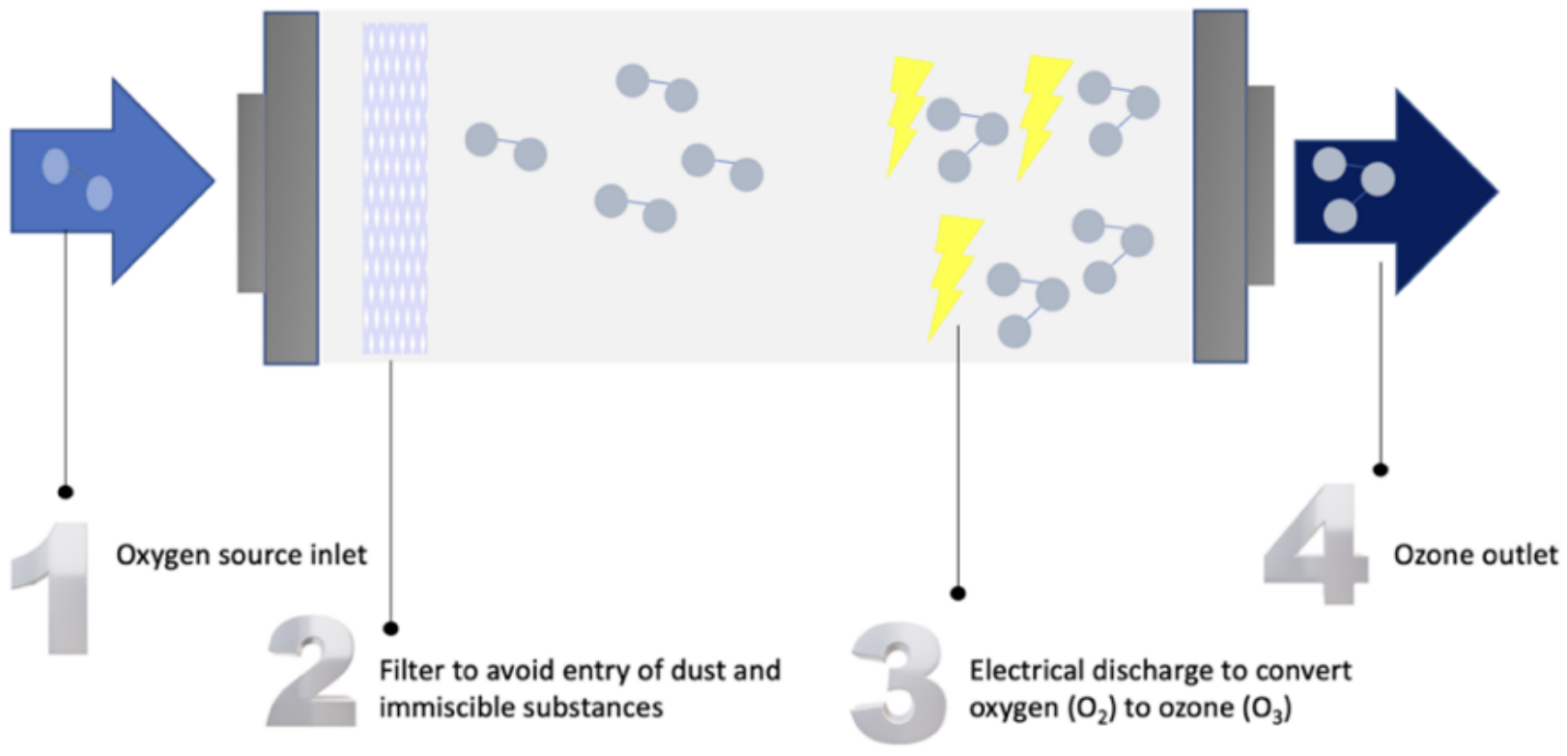
Stages of ozone generation.

WP using ozone is based on on-site ozone generation from pure oxygen, air, or water, as presented in Figure 1. First, the oxygen source is filtered to remove any immiscible substances. Afterward, ozone generators supply energy to oxygen molecules in pure oxygen, air, or water. This causes the split of the oxygen molecule into two single oxygen atoms, which, in turn, combine with the oxygen molecules to form ozone in an endothermic reaction. After that, ozone gas can be pumped into untreated water to oxidize pathogens. It is noteworthy to mention that ozone gas is a stronger oxidizing agent compared to chlorine gas [6].

As a result, WP using ozone generation is effective and its benefits are plentiful. However, the technology behind ozone generation is costly and under intense development. Thus, plenty of research efforts are headed toward finding a cost-effective and reliable method of ozone generation for WP. Consequently, the main contributions of the paper can be summarized as follows:

- Development of a sinusoidal power supply for an ozone generator for WP that is reliable and meets the required properties for ozone generation.
- Conducting a performance assessment on the designed power supply to ensure the reliability of the power supply and provide a comparison between different sinusoidal power supply topologies, including the effect of the filter parameters on the converter gain.

In the upcoming sections, Section 2 surveys the literature. Next, Section 3 describes the different power supply topologies. After that, Section 4 discusses the results. Finally, Section 5 concludes the paper, and proposes prospective future work.

## 2. Related Work

This paper addresses two main research issues, namely ozone chamber models and sinusoidal power supplies, which are surveyed in the following subsections.

### 2.1. Ozone Chamber Model

In the existing literature, different designs considered modeling the ozone chamber using different methods, depending on the input of the ozone chamber, which could be air or oxygen. Aqui-Tapia et al. [7] modeled the ozone chamber as a series RC with the input being either air or oxygen. The capacitor and resistor values were 2.71 nF and 19.16 kΩ, respectively. Moreover, Tang et al. [8] modeled the chamber with two series capacitors having values of 0.157 µF and 0.5248 µF, where back-to-back zener diodes are connected in parallel with the 0.5248 µF capacitor. The zener diodes’ voltage is 2100 V and the input of the chamber is oxygen. On the other hand, Ibarra et al. [9] utilized a parallel RC model, where the input of the chamber can be air, dry air, or oxygen. Finally, using the values of 700 kΩ and 87 pF, Facta et al. [10] created a parallel RC model with the input being either oxygen or air.

As seen in Table 1, the most common model for the ozone chamber is the capacitor in parallel with a resistor. Ideally, the ozone chamber would act as a pure capacitor. In this paper, the parallel capacitor and resistor model is utilized.

### 2.2. Sinusoidal Power Supplies for Ozone Generation

Nassour et al. [11] compared the use of surface and volume dielectric barrier discharge generators utilizing a sinusoidal power supply. The two models were compared in terms of ozone concentration, energy efficiency, and cooling performance. The research showed that the surface discharge model has better ozone generation efficiency.

Further, Gnapowski et al. [12] examined the effect of geometry on the performance of glass dielectric barrier discharges in terms of efficiency, power, and homogeneity. The results showed that homogeneous discharges increase ozone efficiency, decreasing the cost of ozone generation.

**Figure 2:**
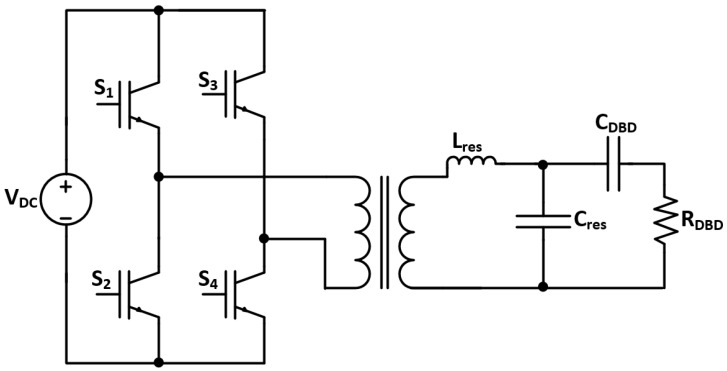
Voltage source full-bridge inverter topology [7].

Aqui-Tapia et al. [7] analyzed the use of inverters for ozone generation. The paper compared two main topologies: a voltage source full-bridge inverter (Figure 2) and a current source full-bridge inverter. It was seen that the decrease in the duty cycle of the modulation signal increases ozone generation, which is due to evading the dissociation of ozone molecules. The voltage source full-bridge inverter provided the highest concentration and efficacy in ozone generation, while the current source full-bridge inverter with closed-loop control provided the highest electrical efficiency.

**Figure 3:**
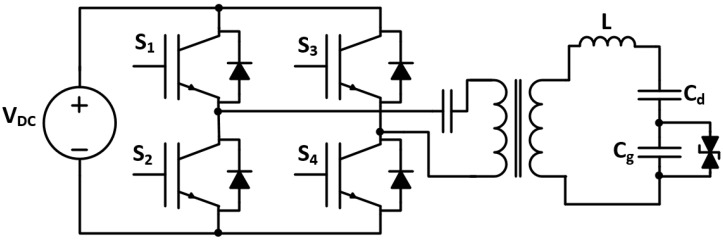
Series-resonant inverter’s wide-frequency topology [8].

Tang et al. [8] examined the utilization of series-resonant inverters in ozone generators with a wide-range frequency model, as shown in Figure 3. The proposed topology allows the use of the ozone generator in a range of working conditions. Simulation and practical results proved the validity of the designed power supply. However, due to designing the model for various conditions, its performance is lower than other power supplies designed for specific conditions.

Additionally, Ibarra et al. [9] tested ozone generation for the WP of a water well in Colombia using a high-frequency, high-voltage flyback inverter. The utilized inverter controlled the generated ozone volume through the duty cycle modulation. The experimental results proved the validity of the prototype for the continuous generation of ozone. The prototype successfully removed all pathogenic micro-organisms in the water and improved the water’s physiochemical properties.

The utilization of a double dielectric barrier discharge chamber for ozone generation was researched by Facta et al. [10]. The ozone generator is operated at atmospheric pressure and is supplied by a high-frequency power converter. Experimental results showed that production levels of 40, 60, and 157 parts per million of ozone were reached from natural air, dry air, and oxygen. The advantage of the design is the low cost, which makes it suitable for low-density applications such as WP at homes.

In addition, Alonso et al. [13] worked on the design of low-power, high-voltage, and high-frequency power supply for ozone generation. The simulation and experimental results showed that the designed converter is effective for low-power output and can be scaled for high-power applications.

Kuwahara [14] reviewed the decrease in the energy consumption in fuel cells used for WP through the bubbling of ozone. The proposed solution is based on water electrolysis, where oxygen and hydrogen are produced. Oxygen is utilized in ozone generation, while hydrogen is utilized in fuel cells for electric power generation. The results proved that this solution provides an alternative method of ozone generation using water rather than air or oxygen tanks.

**Figure 4:**
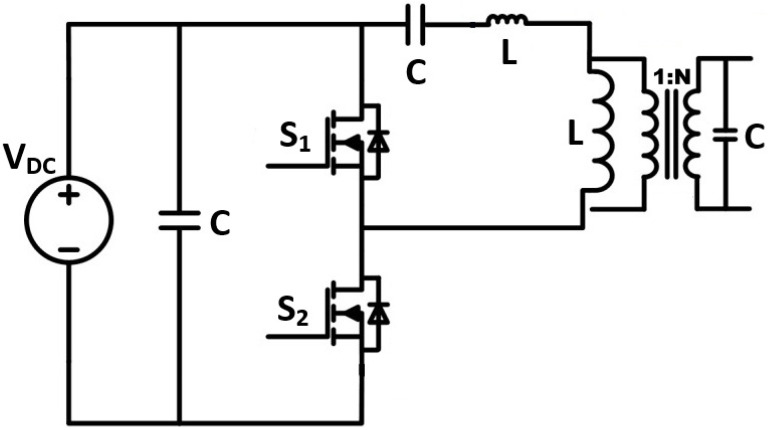
High-voltage half-bridge inverter topology [15,16].

Wellawatta et al. [15] and Neretti and Ricco [16] explored the usage of a high-voltage half-bridge inverter topology, shown in Figure 4. Wellawatta et al. [15] compared the usage of sinsuoidal power supplies compared to pulsed power supplies for ozone generation. It was shown that sinusoidal power supplies produce more ozone due to a larger activation time. In the work by Neretti and Ricco [16], it was shown that such a topology can produce up to 6 kV. However, the main downside of such a topology is the use of a high-frequency transformer, which can be costly.

Table 2 compares some of the different topologies in the literature.

**Figure 5:**
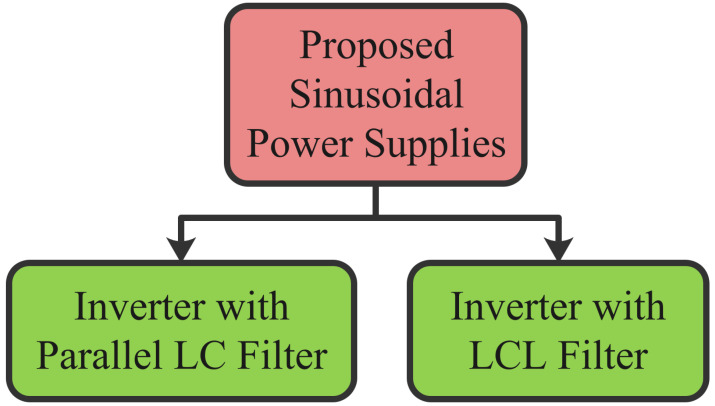
Tree diagram of practical implementation of proposed designs.

Figure 5 displays the tree diagram of the designs that will be presented in this paper. As seen in the figure, two designs will be presented, which are the inverter with a parallel LC filter (Section 3.1 and Section 4.1) and an inverter with an LCL filter (Section 3.2 and Section 4.2). Both designs will be analyzed mathematically, through simulation, and will be implemented practically.

## 3. Power Supply Topologies

As previously mentioned, the parallel ozone chamber model is utilized for the analysis of the power supply topologies due to its simplicity. Moreover, the parallel ozone chamber model has been highly utilized in previous studies since the ozone chamber is a dielectric discharge chamber, which acts as a capacitor in parallel with a resistor with a very high resistance (in the MΩ range). In this study, the values of the capacitor and the resistance are 200 pF and 1 MΩ, respectively.

**Figure 6:**
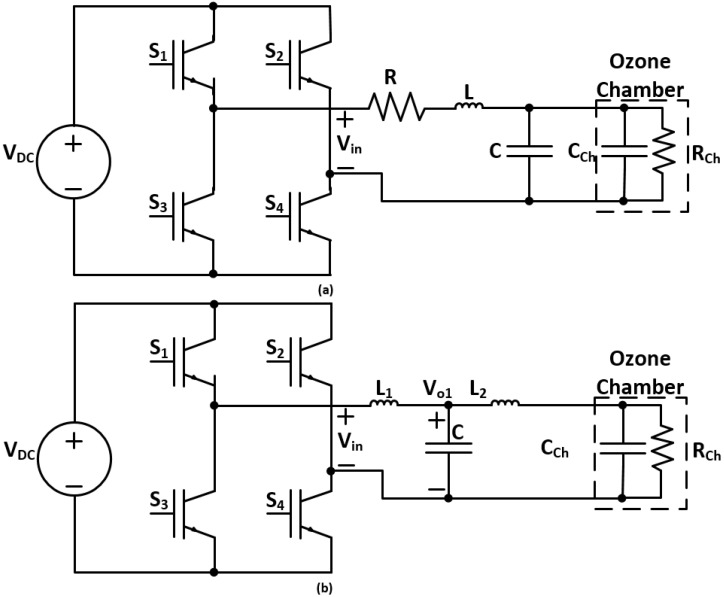
Inverter with (**a**) parallel LC filter and (**b**) LCL filter topologies.

Furthermore, in this paper, two different power supply topologies are analyzed and compared for their feasibility and voltage gain, which are the inverter followed by a parallel LC filter [7], and an LCL filter. The following subsections derive the mathematical models and analyses of the different sinusoidal power supply topologies, as shown in Figure 6.

The advantages of such topologies are the possible modularity in the design, as well as the high gain through the resonance between the L and C passive components. Moreover, the transformerless design decreases the cost, in addition to the low voltage rating of the IGBT switches, which is rated at $V_{dc}$.

### 3.1. Inverter with Parallel LC Filter

Figure 6a provides the schematic diagram for the sinusoidal power supply based on the inverter with a parallel *LC* filter [7]. The derivation for the transfer function and the gain of the parallel *LC* filter is shown below.

Equation (1) provides the calculation of *L* and *C* for the filter.

(1) $$f_{c}=\frac{1}{2\pi \sqrt{LC}}$$

The parallel equivalent impedance ($Z_{eq}$) of the ozone chamber and the resonant capacitor is calculated as shown in (2), where $R_{ch}$ and $C_{ch}$ are the resistance and capacitance of the ozone chamber, respectively.

(2) $$Z_{eq}=\frac{R_{ch}}{1+j\omega R_{ch}\left(C+C_{ch}\right)}$$

Equation (3) highlights the relationship between the input voltage ($V_{in}$) at the terminals of the resonant circuit and the output voltage ($V_{o}$), where $C_{eq}=C+C_{ch}$.

(3) $$V_{o}=\frac{Z_{eq}}{Z_{eq}+R+j\omega L}V_{in}$$

The transfer function (T(jω)) of the parallel *LC* filter gain is defined in (4).

(4) $$\begin{array}{cc} T(j\omega ) & =\frac{V_{o}}{V_{in}}\\ \; & =\frac{R_{ch}}{\left(R+R_{ch}-\omega^{2}R_{ch}LC_{eq}\right)+j\omega \left(L+RR_{ch}C_{eq}\right)} \end{array}$$

Finally, the half-power bandwidth ($\omega_{3dB}$) of the filter is elucidated in (5), where $A_{1}={\left(R_{ch}LC_{eq}\right)}^{2}$, $A_{2}=L^{2}+{\left(R_{ch}C_{eq}\right)}^{2}+2LR_{ch}C_{eq}-2RR_{ch}LC_{eq}-2R_{ch}^{2}LC_{eq}$, and $A_{3}=R^{2}-R_{ch}^{2}+2RR_{ch}$.

(5) $$\omega_{3dB}=\pm \sqrt{\frac{-A_{2}\pm \sqrt{A_{2}^{2}-4A_{1}A_{3}}}{2A_{1}}}$$

**Figure 7:**
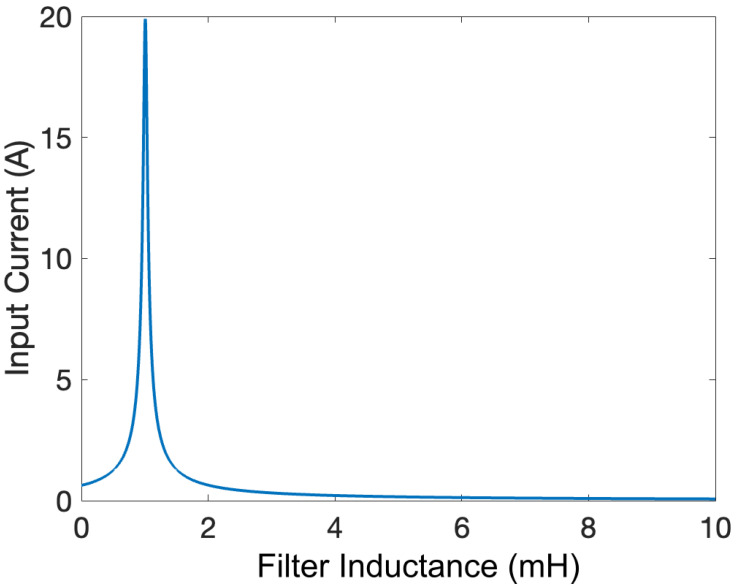
Input current as a function of L for C = 40 nF.

As seen from the mathematical formulation, the quality factor depends on the filter’s *R*, *L*, and *C* values. If a small value of *L* is utilized to achieve a high gain, the input current is high due to the resonance of the *L* and *C* components of the filter, as shown in Figure 7. Moreover, using the filter without a damping resistance causes underdamped oscillations in the output voltage. As a result, a small damping resistance should be added, in addition to the requirement of increasing the value of *L* to limit the input current.

This typology’s main drawback is the gain’s sensitivity to changes in the *R*, *L*, and *C* passive components. The changes in the passive components’ values cause the shifting of the resonant frequency or decrease in the gain, affecting the model’s performance. Thus, sensitivity analysis should be performed to ensure that the model achieves high performance. Due to the small value of the chamber capacitance, it does not affect the model’s sensitivity since the resonant circuit’s capacitor is dominant. Similarly, the resistance of the chamber is high, meaning it can be assumed to be an open circuit and has a negligible effect on the sensitivity of the topology.

**Figure 8:**
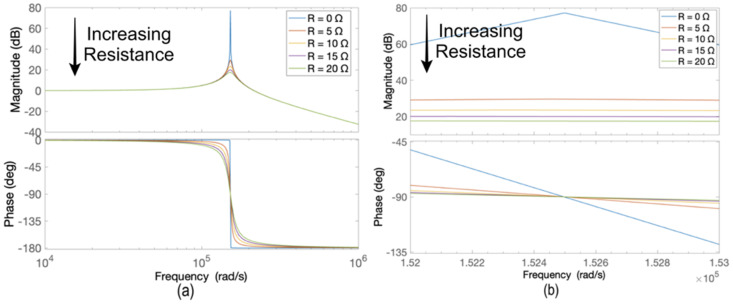
(**a**) Bode plots for sinusoidal power supply based on an inverter with a parallel LC filter and (**b**) zoomed Bode plots for R values from 0 to 20 Ω.

Figure 8 displays the Bode plot of the model for damping resistance values ranging from 0 to 20 Ω, in increments of 5 Ω. As seen from the figure, increasing the value of the resistor from 0 to 5 Ω decreases the gain from 77 dB to 29 dB. Further increases in the resistance cause slight changes in the value of the gain in dB.

**Figure 9:**
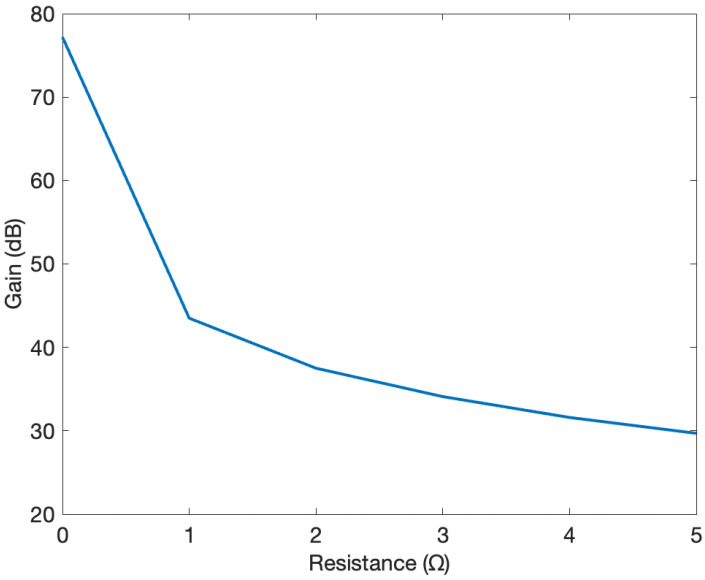
Gain curve for R values from 0 to 5 Ω.

Similarly, Figure 9 provides the maximum gain curve corresponding to the R values in Figure 8. As shown in Figure 9, increasing the resistance causes increasingly smaller drops in the gain. Moreover, it can be deduced that the gain in dB decreases as the resistance increases.

**Figure 10:**
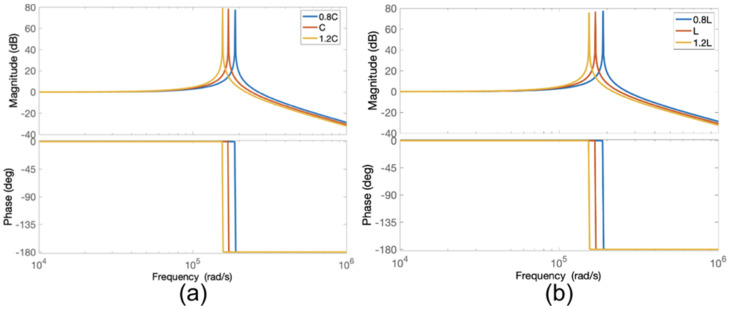
Bode plots for sinusoidal power supply based on an inverter with a parallel LC filter for (**a**) ±20% deviation in C with fixed L and (**b**) ±20% deviation in L with fixed C.

Additionally, Figure 10 illustrates the Bode plots for a ±20% deviation in the C with fixed L values and a ±20% deviation in the L with fixed C values, respectively. As shown in Figure 10, the changes in the L and C values cause a shift in the resonant frequency. Thus, the switching frequency should be controlled to ensure that the maximum gain is achieved from the resonant circuit.

When considering a corner frequency of 25 kHz, an L-value of 1 mH and a C-value of 40 nF should be utilized. Table 3 displays the parameters for the sinusoidal power supply based on an inverter with a parallel LC filter topology.

### 3.2. Inverter with LCL Filter

Figure 6b provides the schematic diagram for the sinusoidal power supply based on the inverter with an LCL filter. The derivation for the transfer function and the gain of the LCL filter is shown below.

Equation (6) calculates the parallel equivalent impedance ($Z_{eq,1}$) of the ozone chamber model, with the series inductance $L_{1}$.

(6) $$Z_{eq,1}=j\omega L_{2}+\frac{R_{ch}}{j\omega R_{ch}C_{ch}+1}$$

The equivalent impedance of $Z_{eq,1}$ and the parallel capacitor of the filter is shown in (7).

(7) $$Z_{eq,2}=\frac{j\omega L_{2}\left(sR_{ch}C_{ch}+1\right)+R_{ch}}{j\omega R_{ch}\left(C+C_{ch}-\omega^{2}L_{2}CC_{ch}\right)+\left(1-\omega^{2}L_{2}C\right)}$$

The gain transfer function (T(jω)) of the power supply based on an inverter with an LCL filter topology is defined in (8), assuming $V_{o1}$ is the voltage across the capacitor of the filter, and $A_{4}=2R_{ch}L_{1}L_{2}^{2}CC_{ch}^{2}$, $A_{5}=4R_{ch}L_{1}L_{2}^{2}CC_{ch}$, $A_{6}=2\left(L_{1}L_{2}^{2}C+R_{ch}^{2}L_{2}^{2}C_{ch}^{2}+R_{ch}^{2}L_{1}L_{2}C_{ch}^{2}\right)+3R_{ch}^{2}L_{1}L_{2}CC_{ch}$, $A_{7}=2\left(R_{ch}L_{1}L_{2}C_{ch}+2R_{ch}L_{2}^{2}C_{ch}\right)+3R_{ch}L_{1}L_{2}C$, $A_{8}=2\left(R_{ch}L_{2}C_{ch}+L_{2}^{2}\right)+3R_{ch}^{2}L_{2}C_{ch}+R_{ch}^{2}L_{1}C_{ch}+R_{ch}^{2}L_{1}C$, $A_{9}=3R_{ch}L_{2}+2L_{2}$, $A_{10}=R_{ch}\left(1+R_{ch}\right)$, $N_{1}=R_{ch}^{2}L_{2}^{2}C_{ch}^{2}$, $N_{2}=2L_{2}\left(R_{ch}^{2}C_{ch}+1\right)$, $N_{3}=2R_{ch}L_{2}$, and $N_{4}=2R_{ch}L_{2}^{2}C_{ch}$.

(8) $$\begin{array}{cc} \; & T\left(j\omega \right)=\frac{V_{o}}{V_{in}}=\frac{V_{o1}}{V_{in}}\frac{V_{o}}{V_{o1}}\\ \; & =\frac{\omega^{4}N_{1}-\omega^{2}N_{2}+R_{ch}^{2}+j\left(\omega N_{3}-\omega^{3}N_{4}\right)}{\left(-\omega^{6}A_{4}+\omega^{4}A_{6}-\omega^{2}A_{8}+A_{10}\right)+j\left(\omega^{5}A_{5}-\omega^{3}A_{7}+\omega A_{9}\right)} \end{array}$$

Finally, the corner frequency ($\omega_{c}$), which is the resonant frequency, can be found as shown in (9).

(9) $$\omega_{c}=\sqrt{\frac{-\left(\frac{L_{2}-L_{1}}{C}-\frac{L_{1}}{C_{ch}}\right)\pm \sqrt{{\left(\frac{L_{2}-L_{1}}{C}-\frac{L_{1}}{C_{ch}}\right)}^{2}+\frac{4L_{1}L_{2}}{CC_{ch}}}}{2L_{1}L_{2}}}$$

In order to obtain good filtering characteristics and have a sinusoidal output at the ozone chamber, $L_{1}$ has to be increased to a high value, which is bulky in size. However, this configuration provides a significantly higher gain compared to the parallel LC filter. In addition, the main limitation of the LCL filter is the requirement to utilize a low-resistance inductor to avoid increased damping. Thus, due to the nature of the required inductor (large inductance and low resistance), its cost will increase. Otherwise, the gain of the LCL filter decreases significantly.

**Figure 11:**
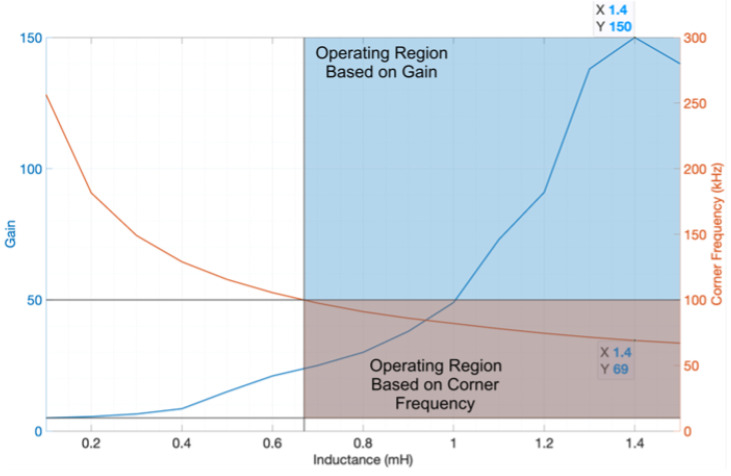
Variation in the gain and corner frequency of the LCL filter as $L_{1}$ changes (blue is the operating region based on gain and orange is the operating region based on corner frequency).

The variation in the value of $L_{1}$ affects the filter’s gain, as shown in Figure 11, which affects the filter’s corner frequency. As a result, to have a feasible switching frequency (between 10 and 100 kHz) and high gain (greater than 10), the operating region is highlighted in the graph. As a result, to maximize the filter’s gain and stay in the operating region, an inductance value of 1.4 mH is selected, corresponding to a switching frequency of 69 kHz, as shown in Figure 11. Table 3 displays the values of the parameters for the sinusoidal power supply based on an inverter with an LCL filter topology.

**Figure 12:**
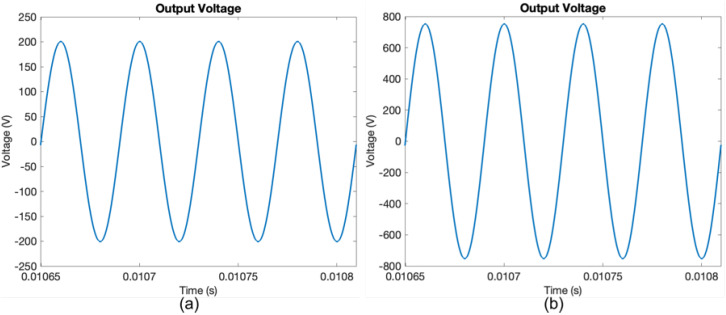
Output voltage waveform for 5 V dc input from simulation for sinusoidal power supply based on an inverter with (**a**) parallel LC filter and (**b**) LCL filter.

Simulink models were utilized to simulate the sinusoidal power supply based on an inverter with a parallel LC filter and an LCL filter. The simulation parameters for the models are seen in Table 3. Moreover, Figure 12 conveys the output voltage for the ozone chamber for the two models from Simulink.

## 4. Practical Implementation

### 4.1. Inverter with Parallel LC Filter

Table 3 provides the values of the parameters for the practical implementation of the parallel LC filter and LCL filter. The damping resistor is assumed to be the internal resistance of the passive components.

**Figure 13:**
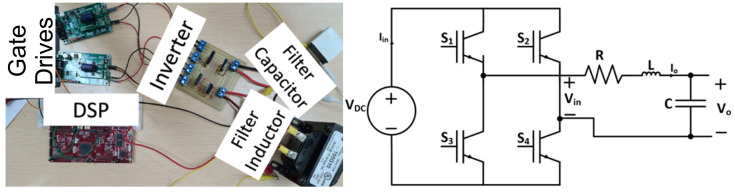
Test rack for practical implementation of the sinusoidal power supply based on an inverter with a parallel LC filter design.

Figure 13 conveys the test rack for practically implementing the sinusoidal power supply based on an inverter with a parallel LC filter design. The switching frequency was set to 25 kHz to follow the same parameters as the simulation.

**Figure 14:**
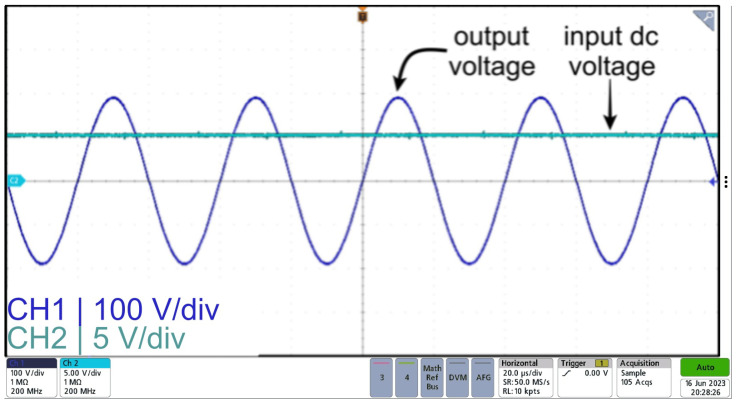
Input voltage (CH2) and output voltage (CH1) waveforms for sinusoidal power supply based on an inverter with a parallel LC filter.

Moreover, Figure 14 displays the input voltage and output voltage of the sinusoidal power supply based on an inverter with a parallel LC filter design. As seen from the figure, a high-frequency sinusoid is achieved, with an approximate gain of 40, when comparing the peak of the output voltage and the dc input voltage, which is in agreement with the results from the simulation.

The voltage amplification of a resonant parallel LC filter can be enhanced by reducing the filter’s inductance, causing a shift in the frequency response magnitude towards higher values. However, this adjustment will lead to an increase in input current, subsequently expanding the circuit’s physical dimensions. Consequently, there exists a trade-off between the size of the power supply and the level of amplification. If the size of the power supply is decreased, the gain of the parallel LC filter would decrease, and, conversely, prioritizing a substantial gain will necessitate a larger power supply. Nevertheless, the practical implementation successfully conforms to the specified gain constraints of the design.

Furthermore, the voltage and current applied to the switches represent the input voltage and input current. Consequently, it can be inferred that the switches are subjected to minimal voltage and current stresses. In contrast, the voltage across the filter capacitor and the current passing through it mirror the output voltage and output current. This implies that the capacitor encounters high voltage stresses, necessitating the use of a capacitor with a high voltage rating. On the other hand, the output current is minimal, resulting in reduced current stresses on the parallel LC filter. Additionally, the voltage stresses on the filter inductor remain low due to the high voltage across the filter capacitor.

In summary, the primary limitation of a sinusoidal power supply utilizing an inverter with a parallel LC filter design is the need for a high-voltage-rated capacitor. Moreover, there exists a trade-off between the size of the power supply and the desired voltage gain.

### 4.2. Inverter with LCL Filter

**Figure 15:**
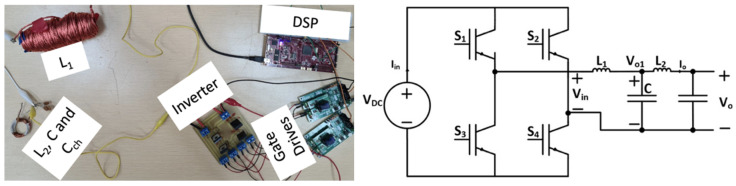
Test rack for practical implementation of the sinusoidal power supply based on an inverter with an LCL filter design.

Another sinusoidal power supply design implemented practically was the inverter with the LCL filter. Table 3 conveys the utilized parameters for the LCL filter design. Figure 15 displays the test rack for the practical implementation of the sinusoidal power supply based on an inverter with an LCL filter design. The load resistor is considered an open circuit for simplicity. However, the load capacitor is considered in the circuit, as it will affect the filter’s corner frequency.

**Figure 16:**
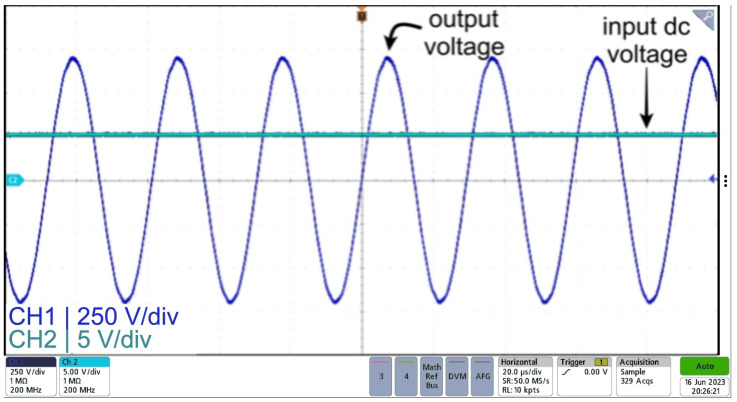
Input voltage (CH2) and output voltage (CH1) waveforms for sinusoidal power supply based on an inverter with an LCL filter.

Also, Figure 16 shows the input voltage, inverter output voltage, and output voltage of the sinusoidal power supply based on an inverter with an LCL filter design. As seen from the figure, a high-frequency sinusoid is obtained with an approximate gain of 150, which is in agreement with the results from the simulation.

Moreover, similar to the topology of the inverter with a parallel LC filter, the voltage and current applied to the switches represent the input voltage and input current. Thus, the switches are subjected to minimal voltage and current stresses. Also, the capacitor encounters high voltage stresses, necessitating the use of a capacitor with a high voltage rating. On the other hand, the output current is minimal, resulting in reduced current stresses on the LCL filter. Additionally, the voltage stresses on the both inductances remain low due to the high voltage across the chamber capacitor.

In summary, the primary limitation of a sinusoidal power supply utilizing an inverter with an LCL filter design is the need for a filter capacitor with a high voltage rating. Moreover, in order to achieve the high gain, $L_{1}$ has to have a low resistance. If an inductor with a high internal resistance is used, the damping of the filter increases, which decreases the gain significantly.

**Figure 17:**
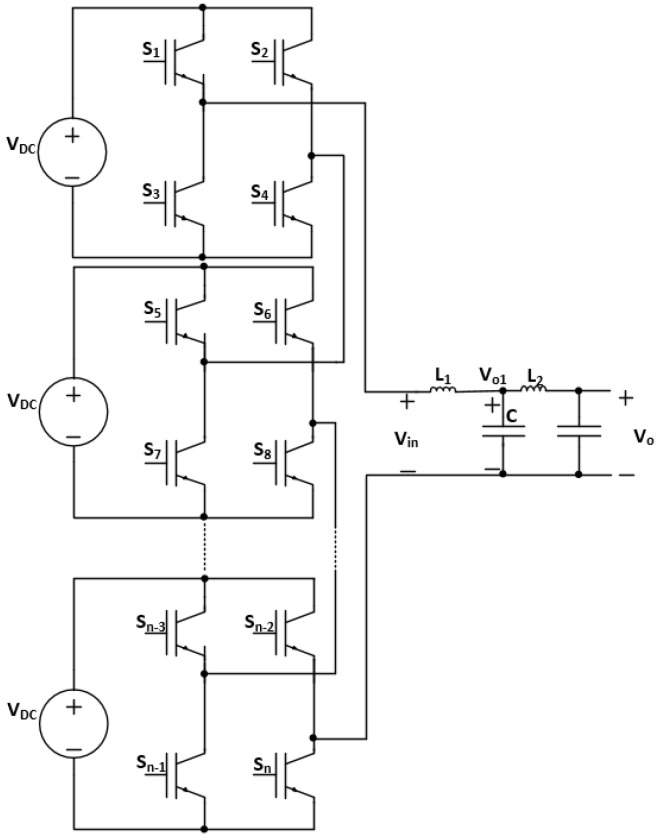
Modular design of sinusoidal power supply based on an inverter with an LCL filter.

Furthermore, it is noteworthy to mention that the sinusoidal power supply design based on an inverter with an LCL filter is a modular design using multiple inverters, as shown in Figure 17. Thus, the total gain of the power supply becomes the gain of a single filter multiplied by the number of inverters.

## 5. Conclusions

In response to the urgent need for clean water access, growing demands for water security, and the global commitment to the SDGs, extensive efforts have been dedicated to advancing WP methods. This study focuses on the development of a modular sinusoidal power supply for an ozone generator used in WP. Two power supply designs were introduced and compared: one featuring an inverter with a parallel LC filter and the other incorporating an LCL filter.

The parallel LC filter offers a modest gain of 40 due to the increased inductance value, which reduces input current. However, when the application can accommodate a higher input current, the gain of the parallel LC filter can be enhanced. Conversely, the LCL filter yields a substantial gain of 150 with an acceptable input current, but it necessitates an inductor with minimal resistance to prevent excessive damping, which could otherwise reduce the filter’s gain significantly.

Subsequently, a performance assessment was conducted to verify the power supply’s efficacy and reliability in delivering the required voltage levels. The results demonstrated that the sinusoidal power supply with the LCL filter met the necessary gain and output voltage criteria.

Future work will revolve around constructing the ozone chamber to evaluate the power supply’s reliability in ozone production, using ozone measurement strips. Additionally, the ozone generator will be employed for water purification, assessing its effectiveness in WP through various water testing methods. Furthermore, machine learning models can be employed to predict the optimal switching frequency and output voltage levels, optimizing ozone generation and enhancing water purity.

## Figures and Tables

**Table 1:** Ozone chamber models.

Reference	Model Structure	Model Values
[7]	Series RC	R = 19.16 kΩ
		C = 2.71 nF
[8]	Series capacitors and	C1 = 0.157 µF
	back-to-back zener diodes	C2 = 0.5248 µF
[9]	Parallel RC	R = 1 MΩ
		C = 100 pF
[10]	Parallel RC	R = 700 kΩ
		C = 87 pF

**Table 2:** Comparison between different topologies in the literature.

Reference & Application	Advantages	Disadvantages
[7] Ozone Generation Based on Parallel LC Filter	High-voltage output Medium power output No ozone dissociation	Large inductance High input current
[8] Ozone Generation Based on Series LC Filter	High power output Wide frequency range	Low efficiency Large inductance
[9] Home Water Treatment Based on Parallel LC Filter	High efficiency Small size	Transformer losses Large inductance
[10] Ozone Generation Based on Parallel LC Filter	High-voltage output High ozone rate	Transformer losses
[13] DomesticWater Treatment Based on Parallel LC Filter	High-voltage output High efficiency	Transformer losses Low power output
[15,16] Ozone Generation Based on Half-Bridge Inverter	High-voltage output High ozone rate	Transformer losses

**Table 3:** Parameters of sinusoidal power supply designs.

Parameter	LCL Filter	Parallel LC Filter
$V_{dc}$	5 V	5 V
Switching Frequency	69 kHz	25 kHz
R	5 Ω	5 Ω
$L_{1}$	1.4 mH	1 mH
$L_{2}$	3 µH	N/A
C	1 nF	40 nF

## Data Availability

The original contributions presented in the study are included in the article, further inquiries can be directed to the corresponding author.
